# Supplementary material for: Predicting visual function by interpreting a neuronal wiring diagram
Source: Nature. 2024 Oct 2;634(8032):113–23. doi: 10.1038/s41586-024-07953-5 (PMC11446822; doi:10.1038/s41586-024-07953-5)
Supplement: Supplementary file 6 — CRF and ERF predictions for individual TmY4 and TmY9 cells. Analogous to Supplementary Data 3, but for TmY target types. Shown are the top four monosynaptic pathways, the strongest pathway passing through each of the top ten intermediary types (ranking from Extended Data Fig. 7), and the trisynaptic pathway Tm1–TmY–Dm3–TmY (see the section entitled Prediction of spatial normalization). [file 41586_2024_7953_MOESM6_ESM.zip › DataS4/TmY9q/720575940613653741.pdf]

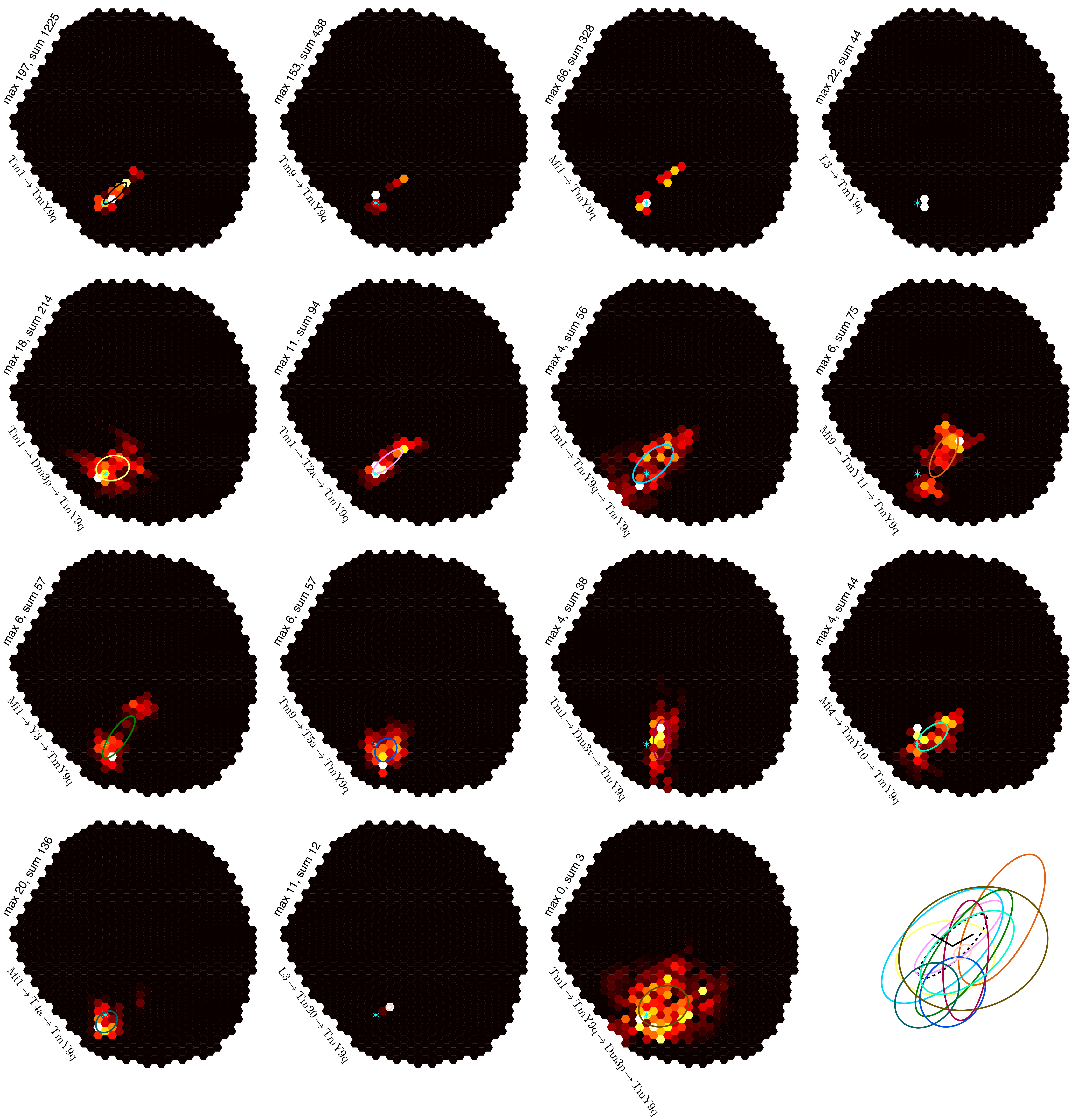

max 197, sum 1225
Tm1 → TmY9q
max 153, sum 438
Tm9 → TmY9q
max 66, sum 328
Mi1 → TmY9q
max 22, sum 44
L3 → TmY9q
max 18, sum 214
Tm1 → Dm3p → TmY9q
max 11, sum 94
Tm1 → T2a → TmY9q
max 4, sum 56
Tm1 → TmY9q → TmY9q
max 6, sum 75
Mi9 → TmY11 → TmY9q
max 6, sum 57
Mi1 → Y3 → TmY9q
max 6, sum 57
Tm9 → T5a → TmY9q
max 4, sum 38
Tm1 → Dm3v → TmY9q
max 4, sum 44
Mi4 → TmY10 → TmY9q
max 20, sum 136
Mi1 → T4a → TmY9q
max 11, sum 12
L3 → Tm20 → TmY9q
max 0, sum 3
Tm1 → TmY9q → Dm3p → TmY9q
